# A qualitative study on the self-management experience and needs of postmenopausal patients with metabolic dysfunction-associated steatotic liver disease from the perspective of active health

**DOI:** 10.3389/fpubh.2026.1828862

**Published:** 2026-07-15

**Authors:** Yuying Yang, Jinjin Cao, Lu Zhang, Jing Liu, Mei Li

**Affiliations:** 1 School of Nursing, Nanjing Medical University, Nanjing, China; 2 Department of Nursing, Nanjing BenQ Medical Center, The Affiliated BenQ Hospital of Nanjing Medical University, Nanjing, China; 3 International Medical Services, Nanjing BenQ Medical Center, The Affiliated BenQ Hospital of Nanjing Medical University, Nanjing, China

**Keywords:** active health, metabolic dysfunction-associated steatotic liver disease, postmenopausal women, qualitative study, self-management

## Abstract

**Background:**

Metabolic dysfunction-associated steatotic liver disease is a prevalent chronic liver disease globally. Postmenopausal women may face a higher risk of developing this disease due to the metabolic changes related to menopausal transition. Although lifestyle adjustments remain the core of the care process, sustained self-management often remains highly challenging. This study, based on the theory of active health, aims to explore the self-management experience of postmenopausal women and the supportive needs.

**Methods:**

A descriptive qualitative design was employed. From August to October 2025, we recruited 16 postmenopausal MASLD patients from a tertiary Grade A hospital in Nanjing. Semi-structured, face-to-face interviews were conducted. Data were independently analyzed by two researchers using thematic analysis.

**Results:**

Sixteen postmenopausal women with MASLD were interviewed. Five themes were generated: (1) Inadequate awareness of MASLD risk; (2) obstacles under the physical and mental burden related to menopause; (3) gaps in the support system; (4) bidirectional effects of emotional experiences on self-management; (5) active health seeking and self-motivation.

**Conclusion:**

Self-management in postmenopausal women with MASLD is shaped by interacting cognitive, physical, emotional, and family factors, while many retain modifiable motivation and initiative. These findings provide valuable implications for development of future interventions targeting this population.

## 1 Introduction

Metabolic dysfunction-associated steatotic liver disease (MASLD; formerly nonalcoholic fatty liver disease, NAFLD) is a common chronic liver disease in clinical practice, belonging to liver diseases related to metabolic disorders. Its characteristic is the accumulation of fat in the liver and at least one metabolic abnormality (1, 2). In 2023, three large multinational liver associations proposed that metabolic dysfunction-associated steatotic liver disease (MASLD) should replace the term NAFLD (3). The prevalence of MASLD among adults is approximately 38%, rising to about 65% in individuals with type 2 diabetes (1).

Gender and age are key determinants of the development and progression of MASLD, however, in women, these factors do not exert simply additive effects. Prior to menopause, estrogen may confer partial protection against hepatic steatosis and fibrosis. Following menopause, declining estrogen levels are accompanied by increases in visceral adiposity, insulin resistance, and lipid dysregulation, which may accelerate MASLD development and progression. Accordingly, postmenopausal women represent a subgroup requiring particular attention (4, 5). A meta-analysis indicated that menopausal status is associated with an approximately 2.4-fold increase in the odds of developing MASLD in women (6), potentially attributable to the loss of the protective effects of estrogen (7). Evidence suggests that effective self-management interventions can enhance self-efficacy in patients with MASLD (8).

Given the absence of specific pharmacological therapies for MASLD at present, self-management has become increasingly central to comprehensive disease control. Cross-sectional survey data from China suggest that the overall level of self-management among patients with MASLD is suboptimal, with notably inadequate disease-related knowledge management (9). As a distinctive physiological-psychological transition, menopause may confer unique experiences and needs for female patients in terms of illness perception, self-efficacy, and social support.

Active Health Theory advocates a shift from reactive, disease-centered care to active, person-centered health management, viewing health as a dynamic capacity rather than a static state. It emphasizes individuals’ agency in recognizing health risks and the reversibility of disease through lifestyle change, translating knowledge into feasible everyday actions, and sustaining behaviors through self-monitoring and ongoing adjustment with support from the family, community, and healthcare system. It also underscores the importance of early intervention and continuous lifestyle optimization to reduce reliance on medical treatment. From this perspective, effective self-management in MASLD is not merely the acquisition of knowledge, but the ability to initiate and maintain health behaviors within real-life contexts (10, 11).

Although previous qualitative studies have explored aspects such as weight management among patients with MASLD, most have focused on the general population (12). Attention to the barriers and needs encountered by postmenopausal patients when managing their condition within the family context remains insufficient. To address this gap, the present study focuses on postmenopausal patients with MASLD using semi-structured interviews. Guided by Active Health Theory, we aim to gain an in-depth understanding of their lived experiences of self-management, key barriers, and support needs, thereby providing evidence to inform the development of more targeted, feasible family-support strategies and clinical interventions.

## 2 Materials and methods

### 2.1 Study design

A descriptive qualitative research design was employed in this study (13). Guided by the Active Health Theory, semi-structured interviews were employed to conduct in-depth investigations into the self-management experiences and needs of postmenopausal patients with MASLD. To ensure methodological rigor and trustworthiness, this study adhered to the Consolidated Criteria for Reporting Qualitative Research (COREQ): 32-Item Checklist to guarantee research design quality (14). The current study was approved by the Ethical Review Authority (Ethical Approval Number: 2025-KL033).

### 2.2 Participants

Participants were selected through purposive sampling between August and October 2025 from the Health Management Center of a single Grade III Class A hospital in Nanjing, recruiting postmenopausal patients with MASLD. A total of 16 participants were ultimately interviewed and coded as P1-P16.

#### 2.2.1 Inclusion criteria

(1) Postmenopausal women; (2) a confirmed diagnosis of MASLD (based on imaging examinations such as ultrasound or CT indicating fatty liver, or in conjunction with previous clinical diagnostic records); (3) willingness to participate in this study and to provide qualitative data related to self-management; (4) signed informed consent and willingness to provide necessary personal and health-related information; (5) ability to understand and communicate effectively in Chinese.

#### 2.2.2 Exclusion criteria

(1) Presence of significant cognitive impairment or severe mental disorders that would preclude effective participation in the interview; (2) concurrent participation in other interventional studies that may influence self-management behaviors or outcome assessment (e.g., systematic interventions involving medication, diet, or exercise); (3) language communication barriers that prevent completion of the interview in Chinese.

### 2.3 Data collection

The semi-structured interview guide was developed by the research team according to the study aim and Active Health Theory, with reference to previous literature on MASLD self-management, menopause-related health changes, and qualitative research in chronic disease management. The guide was designed to explore participants’ understanding of MASLD, perceived relationship between menopause and MASLD, daily management practices, barriers and facilitators, sources of support, emotional experiences, self-monitoring behaviors, and expectations for future care. After several rounds of team discussion, the wording and sequence of questions were refined to ensure that they were open-ended, understandable, and suitable for postmenopausal women. With participants’ informed consent, two researchers (both were postgraduate students majoring in nursing) with qualitative research training conducted semi-structured interviews in a private and quiet room. Interviews were audio-recorded using a digital recorder with participants’ permission and then transcribed verbatim. The interview duration was approximately 20–40 min. Field notes were taken during/after interviews focusing on non-verbal cues and contextual information. A semi-structured interview schedule was used as a loose guide during interviews (Table 1). The research adhered to strict ethical principles, including voluntary participation, anonymity, and confidentiality. Participants were explicitly informed of their right to withdraw from the study at any time without repercussions.

**Table 1:** Interview guide.

Interview questions
How do you understand your diagnosis of fatty liver disease? In your view, what is the relationship between menopause and fatty liver disease?
To manage fatty liver disease and overall health, what efforts have you currently made in terms of diet, physical activity, daily routines, and emotional regulation?
What are the greatest difficulties or challenges you have encountered during the health management process?
During this process, what types of support or barriers have you experienced from family, friends, colleagues, or the environment (such as family atmosphere, work environment, community, and healthcare resources)?
Which physical signs or indicators do you usually monitor to understand your health status (e.g., body weight, waist circumference)? Do you undergo regular follow-up examinations? When communicating with physicians, do you tend to ask questions actively or mainly follow medical advice?
What motivates you to continue with health management? What are your future management goals and expectations, and what additional information, skills, or support do you need to enhance your confidence and capacity?

### 2.4 Data analysis

This study analyzed interview data using thematic analysis, with Active Health Theory providing an analytic lens. With participants’ consent, all interviews were digitally recorded and transcribed verbatim as soon as possible after completion; transcripts were checked against the original recordings to ensure accuracy. Data analysis followed Braun and Clarke’s approach (15). First, researchers repeatedly read the transcripts to become familiar with the content and documented preliminary reflections. Next, meaningful segments of text were systematically coded; through constant comparison, codes were refined and organized into broader categories, from which preliminary themes were developed. Finally, themes were reviewed and revised through team discussions to clarify their boundaries and define their meanings. NVivo 15 was used to support data management and coding. All coding was independently completed by two authors and then compared to establish a preliminary coding framework. Any discrepancies were resolved through team discussion and, when consensus could not be reached, adjudicated by a third researcher. Findings are presented descriptively and supported by representative quotations. Table 2 provides an example of the data analysis process.

**Table 2:** An example of the data analysis process.

Meaning unit	Summarized meaning unit	Preliminary code	Subtheme	Theme
“It does not really have much impact. At this age, everyone has something wrong. I just let it take its course.”	Patient perceives MASLD as a minor, age-related issue and sees no need for active management.	Minimizing severity or normalizing with aging	Insufficient perceived seriousness of the disease	Inadequate awareness of MASLD risk
“Fatty liver eventually turns into liver cancer, right?”	Patient holds oversimplified and extreme beliefs about MASLD outcomes due to limited knowledge.	Misconceptions about disease progression	Limited knowledge of the disease	Inadequate awareness of MASLD risk
“Those things online are too complicated, and some of them even contradict each other. I cannot understand them, and I do not want to read them.”	Patient struggles to interpret inconsistent online information and disengages from learning.	Difficulty interpreting health information or Information overload	Limited access to information	Inadequate awareness of MASLD risk
“Exercise—there’s no point talking about it now. My knees and lumbar spine are not good, so I do not exercise much. With osteoporosis, there are many activities I do not dare to do.”	Comorbidities related to menopause and fear of injury restrict exercise participation.	Physical limitations and fear reduce exercise	Physical and psychological limitations to exercise	Obstacles under the physical and mental burden related to menopause
“Healthy food often does not taste good… But when I come across fried or sweet foods, I still want to eat them, so it’s difficult.”	Patient knows dietary restraint is needed but finds palatable foods hard to resist.	Low dietary self-control or preference for palatable foods	Internal and external barriers to dietary control	Obstacles under the physical and mental burden related to menopause
“I sit for long periods—five or six hours a day playing on my phone…”	Established sedentary lifestyles become deeply rooted and resistant to change.	Habitual sedentary behavior	Entrenched unhealthy lifestyle patterns	Obstacles under the physical and mental burden related to menopause
“My family does not particularly support me exercising…”	Limited family support and differing household habits hinder sustained self-management.	Lack of family support as a barrier	Barriers from spouses and family members	Gaps in the support system
“First, there’s no time (for exercise)—I have to pick up the child after school and help with homework…”	Caregiving and household responsibilities create time/energy conflicts that crowd out self-care.	Caregiving role strain or Time conflict	Challenges of intergenerational roles and responsibilities	Gaps in the support system
“No one manages it, and the doctor did not say much… it’s all up to me to figure out adjustments.”	Patient experiences minimal follow-up and insufficient individualized guidance from doctors.	Lack of tailored guidance	The lack of interaction between doctors and patients	Gaps in the support system
“I’m a fairly optimistic person… Fatty liver is like a reminder to take health more seriously… overall my mood is quite positive.”	Positive affect supports adherence and reframes illness as motivation for change.	Positive emotions facilitate behavior change	A positive mindset facilitates behavioral adjustment	Bidirectional effects of emotional experiences on self-management
“I worked out at the gym for two or three months—my weight did not go down… That experience really discouraged me.”	Failed weight-loss attempts reduce confidence and weaken motivation to persist.	Discouragement after failed efforts	Negative emotions intensify health depletion	Bidirectional effects of emotional experiences on self-management
“(After menopause) I supplement with some legumes and things like milk.”	Patient actively adopts self-initiated strategies to improve health.	Take active measures for self-management	Self-adjustment to adapt to the illness	Active health seeking and self-motivation
“Every year at my physical exam, I compare what’s different from last year…”	Patient uses check-up results or weight changes to track status and adjust behaviors.	Monitoring outcomes to guide adjustments	Implementation of self-monitoring	Active health seeking and self-motivation
“I hope the plan can be simple and practical, not too complicated—like telling me how to cook home dishes with less oil and salt, and what exercises I can do at home.”	Patient wants individualized, feasible, step-by-step guidance that fits daily life.	Need for tailored, actionable self-management plan	Demand for personalized health management	Active health seeking and self-motivation

## 3 Results

A total of 16 postmenopausal women with MASLD were included in this study. Participants ranged in age from 53 to 70 years, with a menopause age between 45 and 55 years. All participants had experienced natural menopause and were urban residents; most were married and lived with their spouses or family members. Most participants were retired, and educational attainment varied from primary school or below to college level or above. Detailed demographic and clinical characteristics are shown in Table 3. Based on the analysis, five core themes and 14 subthemes were identified, reflecting patients’ experiences (Table 4). The core themes and subthemes are detailed below.

**Table 3:** The demographic and clinical characteristics of the participants.

Characteristics	Participants, *n* (%) or range
Age, years	53–70
Age at menopause, years	45–55
Type of menopause	Natural menopause 16 (100.0)
Marital status	Married 15 (93.8)
	Widowed 1 (6.3)
Education level	Primary school or below 2 (12.5)
	Junior high school 1 (6.3)
	Senior high school/technical secondary school 9 (56.3)
	College/Bachelor’s degree or above 4 (25.0)
Employment status	Retired 16 (100.0)
Place of residence	Urban 16 (100.0)
Living arrangement	Living alone 2 (12.5)
	Living with spouse 8 (50.0)
	Living with children 1 (6.3)
	Living with spouse and children 5 (31.3)
Main comorbidities*	Hypertension 8 (50.0)
	Diabetes mellitus 3 (18.8)
	Osteoporosis 4 (25.0)
	Depression 1 (6.3)
	Coronary heart disease 2 (12.5)
Body mass index, kg/m^2^	21.05–31.24
Waist circumference, cm	73–97
Smoking history	0 (0.0)
Alcohol consumption history	0 (0.0)
Exercise frequency	Almost no exercise 5 (31.3)
	1–2 times/week 4 (25.0)
	3–4 times/week 2 (12.5)
	≥5 times/week 5 (31.3)
Main reason for exercise limitation	Joint pain 4 (25.0)
	Lack of time 8 (50.0)
	Dislike exercise 2 (12.5)
	No limitation 2 (12.5)
Family support level	Strong support 8 (50.0)
	Moderate support 7 (43.8)
	No support 1 (6.3)
Perceived need for MASLD treatment	Necessary 3 (18.8)
	Not necessary 13 (81.3)

Participants could have more than one comorbidity.

**Table 4:** The main themes and subthemes identified from the interviews.

Themes	Subthemes
Inadequate awareness of MASLD risk	Insufficient perceived seriousness of the disease
	Limited knowledge of the disease
	Limited access to information
Obstacles under the physical and mental burden related to menopause	Physical and psychological limitations to exercise
	Internal and external barriers to dietary control
	Entrenched unhealthy lifestyle patterns
Gaps in the support system	Barriers from spouses and family members
	Challenges of intergenerational roles and responsibilities
	The lack of interaction between doctors and patients
Bidirectional effects of emotional experiences on self-management	A positive mindset facilitates behavioral adjustment
	Negative emotions intensify health depletion
Active health seeking and self-motivation	Self-adjustment to adapt to the illness
	Implementation of self-monitoring
	Demand for personalized health management

### 3.1 Theme 1: inadequate awareness of MASLD risk

#### 3.1.1 Subtheme 1: insufficient perceived seriousness of the disease

Many participants did not regard MASLD as a “real illness” in the absence of obvious symptoms, leading them to dismiss the need for active management. This attitude overlooks the risks associated with MASLD progression, resulting in a limited understanding of the necessity for regular monitoring and lifestyle modification. Consequently, opportunities for timely early intervention may be missed. “I do not think it’s serious. As we get older, it’s normal to have these minor issues. I do not really pay much attention to it.” (P12). “I do not really care about these things. What even is fatty liver? Isn’t it just a bit more oil? If you control your diet a little, that’s enough. It’s not really a disease, and there’s no medicine for it anyway. Fatty liver does not actually need treatment.” (P4). Similarly, “I do not have any symptoms. I eat well, sleep well, everything is normal, so I do not take it seriously. I’d only go to the hospital if it became serious.”(P10) In addition, some participants underestimated their own risk by comparing themselves with others around them. “A lot of people around me have it and they are doing just fine. I do not feel uncomfortable, so I do not think it’s something to worry about.”(P6).

#### 3.1.2 Subtheme 2: limited knowledge of the disease

Some participants demonstrated a limited understanding of the etiology, progression, and potential long-term consequences of MASLD. This was reflected in extreme assumptions about outcomes, simplistic attributions of causality, and little effort to update their knowledge. Such cognitive biases may hinder timely and effective self-management. “Fatty liver eventually turns into liver cancer, right?” (P1). “I think my fatty liver came later because I ate too much oil. Back then, I always poured in a lot of oil and liked fried eggs. Then it showed up on the check-up—I never had it before.” (P11). “I think eating too much and not exercising causes fatty liver. But some thin people have it too, so I cannot really make sense of it.” (P13).

#### 3.1.3 Subtheme 3: limited access to information

With the expansion of internet use, postmenopausal women with MASLD obtained disease information through multiple channels; however, both access to information and the ability to interpret it were frequently perceived as major barriers. Although online health information is abundant, much of it is either difficult to understand or lacks credibility, and is often presented without systematic, evidence-based support. These limitations may contribute to inaccurate beliefs about MASLD management or the neglect of clinically important recommendations. “Those things online are too complicated, and some of them even contradict each other. I cannot understand them, and I do not want to read them.” (P6). “My sister sends me some materials. I usually judge by gut feeling—anything that sounds too absolute or claims to cure everything must be fake.” (P7). In addition, some participants faced practical barriers related to healthcare processes and difficulties using digital tools. “It’s hard to go to the hospital now. A lot of things require using a computer, and older people like us aren’t good at that. Even making an appointment is inconvenient.” (P1).

### 3.2 Theme 2: obstacles under the physical and mental burden related to menopause

#### 3.2.1 Subtheme 1: physical and psychological limitations to exercise

Postmenopausal women with MASLD commonly reported osteoarticular problems and chronic conditions that directly constrained both their capacity and willingness to exercise. As a result, many experienced a dilemma of “willing but unable,” or “being afraid to be active,” due to objective health limitations. “Exercise—there’s no point talking about it now. My knees and lumbar spine are not good, so I do not exercise much. With osteoporosis, there are many activities I do not dare to do.” (P1). “When my lower back hurts, I cannot walk, so it affects exercise.” (P11). In contrast, some participants acknowledged declining stamina and joint discomfort yet attempted to compensate by increasing household tasks, treating domestic work as an “acceptable form of activity.” “I used to like cycling when I was younger, but now I’m older and my knees aren’t good, so I do not want to push myself. I look after the kids, cook, and do housework at home—that counts as exercise.” (P6). A small number of patients may set limits for themselves, and this negative mindset may lead them to give up trying new health-management behaviors, thereby adversely affect health outcomes. “I always feel that strength training is something young people do.” (P12).

#### 3.2.2 Subtheme 2: internal and external barriers to dietary control

Dietary control involved a dual set of challenges. Internal barriers were linked to sustained difficulty resisting appetitive, palatable foods, whereas external barriers arose from family dietary preferences and social pressures. Some participants were primarily influenced by personal taste and the appeal of high-energy foods. Even when they understood the importance of a lighter diet, they still struggled to resist fried foods and sweets that were perceived as “tasty but unhealthy.” “Healthy food often does not taste good, and persistence is important. But when I come across fried or sweet foods, I still want to eat them, so it’s difficult.” (P5). “I used to love sweets. I eat fewer sweets now, but my family all knows I like cakes.” (P1). Others were more affected by social contexts—such as family gatherings or social engagements—where they found it difficult to maintain dietary restraint and relaxed dietary standards to avoid appearing discourteous. “When the family gets together, there’s always plenty of rich dishes, and it’s hard to avoid completely.” (P14). “Especially at friends’ gatherings, the food is quite oily and salty. Everyone is happy, and I do not want to spoil the mood.” (P15). Collectively, these accounts indicate that dietary control requires more than simply “knowing one should restrain”; it entails continual negotiation between personal preferences and social expectations.

#### 3.2.3 Subtheme 3: entrenched unhealthy lifestyle patterns

Some participants described long-standing lifestyle habits that were minimally guided, largely passive, and difficult to change. “I eat scallions and garlic every day because I think they are good for the liver. When the doctor said I had fatty liver, I thought, well, these foods can detoxify anyway, so it should be fine.” (P8). “I sit for long periods—5 or 6 h a day playing on my phone. Others might think that’s tiring, but I do not feel tired, and I do not have any back pain.” (P10). In addition, although some participants were relatively cautious with main meals, they reported limited portion control for foods perceived as “healthy,” such as fruit. “But I really love fruit. In summer, I cannot control myself with watermelon and grapes.” (P12).

### 3.3 Theme 3: gaps in the support system

#### 3.3.1 Subtheme 1: barriers from spouses and family members

Several participants perceived limited family support, and differences in diet or lifestyle within the household could become direct barriers, making it difficult to sustain healthy eating. “My family does not particularly support me exercising. The food my family eats is sometimes quite oily, so I cook less of it and try to control it. In reality, it’s mostly that I eat more healthily myself and manage my own diet.” (P13). “Mainly I do not have enough time—I need to take care of my granddaughter and my husband, and sometimes I cannot take care of myself. Also with diet, I know I should eat lightly, but the younger people at home like stronger flavors, and it’s hard not to be influenced.” (P16).

#### 3.3.2 Subtheme 2: challenges of intergenerational roles and responsibilities

For postmenopausal women, family responsibilities and role expectations frequently emerged as key determinants of health management. Many participants faced increasing household burdens and often sacrificed their own time and energy while caring for children or grandchildren. This was reflected not only in time conflicts, but also in differences in dietary practices and lifestyle routines. These intergenerational demands underscore the challenge of balancing family roles with sustained health management after menopause. “First, there’s no time (for exercise)—I have to pick up the child after school and help with homework at night. Second, my body is not like when I was younger; I get tired as soon as I move a little. And I’m not even sure exercise would help.” (P6). “These past 10 years I’ve mainly been taking care of kids, and it really affected my exercise. A lot of times I wanted to go work out, but I had to pick them up after school, so it got delayed.” (P8). “There are a lot of things at home, especially taking care of my grandson. Sometimes he’s very active, and I have to look after him, play with him, and feed him, so there’s really not much time for exercise. There’s no time to arrange other activities. Especially when he goes to bed late, I’m even more tired and do not have the energy to exercise.” (P13). “I do not like overly complicated diets. Recently I’ve been taking krill oil, which is supposed to be good for blood vessels in older people. I usually cook for myself with lighter flavors, but when I’m taking care of my granddaughter, she likes fried chicken and barbecue, and sometimes I eat along with her.” (P16).

#### 3.3.3 Subtheme 3: the lack of interaction between doctors and patients

Many participants expressed distrust or dissatisfaction with medical advice, perceiving the recommendations as overly brief and insufficiently individualized. “I do not really follow the doctor’s arrangements; the advice may not suit me.” (P2). “Yes, I ask the doctor—I definitely do. If I do not ask, the doctor will not mention it actively. I ask what I should pay attention to regarding fatty liver and whether medications might affect the liver.” (P7). “No one manages it, and the doctor did not say much. After the physical exam, no one asked about it—it’s all up to me to figure out adjustments.” (P11). Some participants therefore turned to online information or advice from friends. While this broadened the range of perspectives, it also carries risks of misinformation or advice without a sound scientific basis. “Doctors usually just prescribe medication and finish in a few sentences—they do not say much. When I had liver function tests, my bilirubin was high, so I asked the doctor and he said it was fine. But when I looked it up, I felt high bilirubin is not good for the liver. I search online, and sometimes I listen to traditional Chinese medicine lectures—livestreams talk about how to regulate the body. Sometimes I believe some of it and buy products—not necessarily supplements; sometimes medications too.” (P3). “If it’s convenient, I’ll ask, but I cannot fully trust it. I also look things up online—Baidu and so on—and compare information from multiple sources.” (P9).

### 3.4 Theme 4: bidirectional effects of emotional experiences on self-management

#### 3.4.1 Subtheme 1: a positive mindset facilitates behavioral adjustment

This study found that some participants maintained relatively positive emotional orientations, which helped transform emotional fluctuations into motivation for self-management. This strengthened willingness and self-efficacy to follow medical advice and sustain lifestyle changes, thereby supporting continuous and gradual optimization and consolidation of behaviors such as diet and physical activity. “I’m not especially anxious, but I do feel pressure. I used to think I was quite healthy, but now that I suddenly have a chronic condition, I feel I cannot be careless anymore. I remind myself to keep adjusting, but I do not worry about it every day.” (P14). “I’m a fairly optimistic person. I feel that as long as I keep it under control, it will not be a big problem. Fatty liver is like a reminder to take health more seriously. I feel better emotionally after exercising, so overall my mood is quite positive.” (P15). “I would not say I’m anxious. I think I need to stay even-tempered. The doctor said if I control it, it’ll be fine, so I do what he said. As long as it does not worsen, I feel reassured.” (P16).

#### 3.4.2 Subtheme 2: negative emotions intensify health depletion

Different forms of negative emotional experiences collectively weakened participants’ motivation to sustain lifestyle changes to some extent. “I’m not particularly anxious, but it’s always on my mind. I keep thinking, ‘How did I end up with fatty liver? I never had it before.’ Every time the doctor says I have fatty liver during a check-up, I feel a bit bothered.” (P11). “After menopause, it’s like having some menopausal symptoms—feeling hot, hot flashes. I might be more irritable, with some emotional fluctuations. My metabolism has slowed down too.” (P3). Some participants emphasized that repeated experiences of “losing weight only to gain more” undermined confidence. “My fatty liver developed later, mainly because of weight gain. I used to be 60 kg and tried to lose weight, but my weight went up to over 70 kg. After dieting, it actually became even higher. I worked out at the gym for two or 3 months—my weight did not go down; it increased. That experience really discouraged me.” (P8). Others were more preoccupied with body image changes and social comparison, gradually developing shame and avoidance toward their bodies. “My belly has gotten bigger, like I’ve gained a lot of fat there. Over these years, I’ve hardly dared to look in the mirror. Clothes do not look good, and especially when I see young people dressed slim, I feel very uncomfortable.” (P10).

### 3.5 Theme 5: active health seeking and self-motivation

#### 3.5.1 Subtheme 1: self-adjustment to adapt to the illness

Most participants actively sought to improve health by using dietary supplements, exchanging health information, and developing personal exercise plans. This active orientation not only supported MASLD improvement but also enhanced overall engagement with life. “(After menopause) I supplement with some legumes and things like milk.” (P3). “I often communicate with others to see what they’ re doing and obtain information that’s useful for me.” (P5). “I’ve seen friends with chronic diseases who take medication every day, and their quality of life declines a lot—I do not want that. I want to reduce the severity of fatty liver through exercise and dietary control, ideally make it go away.” (P15). “No one exercises with me, so I signed up for classes so I have companionship.” (P9). Some participants also designed gentler and more feasible activities tailored to their age and joint status, integrating them into daily routines rather than simply copying younger habits or other people’s exercise patterns. “I walk less now, so I came up with a method: I stand by the window and do thousands of heel raises every day. It keeps me active without hurting my knees—it’s very suitable. At this age, intense exercise is not appropriate. I just find small activities that work, and then it gets better. I find it quite remarkable. I think these popular, folk experiences can also be shared with you for everyone to understand—very intense exercise does not necessarily work better.” (P2). Observing improvements in others also served as a motivator, prompting initiation and sustaining of self-management behaviors. “When I saw overweight people run and walk every day and their fatty liver improved, I decided that when the weather cools down I’ll walk for an hour after dinner every day. Walking is better than not moving.” (P10).

#### 3.5.2 Subtheme 2: implementation of self-monitoring

Most participants used objective indicators as the primary basis for self-monitoring, particularly health check-up results and changes in body weight, and made short-term dietary and lifestyle adjustments accordingly. “Every year at my physical exam, I compare what’s different from last year. I do not pay attention to waist circumference, but if my weight is higher, I eat less. Recently I noticed it went up a bit, so I started eating less.” (P2). “I pay attention to my weight—if it’s gone up again, then I need to be careful. For example, when I go to Hainan, I eat, drink, and sleep, and do not do anything, and my weight shoots up. When I come home and start doing things again, it goes down. So I pay attention.” (P4). Conversely, some participants relied more on subjective sensations and day-to-day cues to monitor their physical state. “Although I do not weigh myself often, I pay attention to whether my clothes feel tight or whether I get tired easily—these bodily signals are things I notice.” (P15).

#### 3.5.3 Subtheme 3: demand for personalized health management

Postmenopausal women reported a strong need for personalized MASLD self-management plans, while also facing multiple challenges in implementing such plans. Many participants hoped for simple and practical guidance rather than complex and difficult-to-understand medical instructions. “Online publicity says fatty liver is not a disease, but I do not agree. Medicine is progressing, and public messaging should keep up. Fat accumulating in the liver must affect liver function, so it has to be taken seriously.” (P2). “I hope the plan can be simple and practical, not too complicated—like telling me how to cook home dishes with less oil and salt, and what exercises I can do at home. Ideally it should not cost a lot of money or take too much time.” (P4). “What I need most is someone to tell me the details—how many times a week should I exercise, and for how long each time? What should I eat, and how much? Are there simple recipes or exercise videos I can follow? If professionals could provide regular guidance or online Q&A, that would be even better.” (P12). “When you say a ‘plan,’ do you mean a schedule that’s easier for us to implement? My biggest confusion about diet is that I want to eat healthily, but in real life there are always times when I crave food or want convenience. In those moments, roughly how much flexibility is acceptable without affecting health? For exercise, my knees hurt sometimes, so jumping or very intense movements do not work—I’d prefer something easier but truly effective if I stick with it, ideally with knee-friendly movements. And emotionally—chronic disease is exhausting. Sometimes I get annoyed and irritable and think, ‘Why do I have to watch this and that again?’ If there were someone to talk with, or a doctor could give simple advice, it would feel easier to persist.” (P14). “For example, regarding food, tell us which foods we can eat and which we should limit, and how to cook in a healthier way. I also hope there are exercises suitable for people our age. For someone like me who does not like exercise, if there are simple methods that are easy to maintain, that would be better.” (P16).

## 4 Discussion

### 4.1 Strengthen self-management in postmenopausal patients with MASLD and improve active health assessment

This study showed a polarized pattern of risk perception among postmenopausal patients with MASLD, with some viewing fatty liver disease as a common phenomenon associated with age and others directly equating it with severe outcomes such as liver cancer. This pattern is consistent with findings from Zhou and colleagues in a Chinese population, showing that overall self-management among patients with MASLD is low and that the lowest scores occur in the domain of disease knowledge management (9). A study in patients in the United States by Tincopa and colleagues similarly found that inadequate understanding of MASLD weakens motivation for behavior change (16). Active Health Theory suggests that individuals are more likely to shift from passive medical adherence to sustained active health behaviors only when they adequately perceive health threats, understand that interventions can reverse risk trajectories, and recognize their own central role in maintaining health (17, 18). Therefore, in postmenopausal populations with MASLD, correcting insufficient risk perception should be treated as a key lever to promote self-management and to foster an appropriate sense of urgency.

In addition, this study further found that the process of obtaining health information can itself amplify the insufficient risk perception described above. This reflects both individual limitations in health information literacy and close links to the realities of the healthcare context in China. In a setting where gaps remain between the institutional design of tiered healthcare delivery and actual health seeking behaviors, patients may bypass primary care and seek higher-level hospitals directly because of concerns about the capacity of primary services (19). The concentration of large numbers of patients in higher-level hospitals further increases outpatient and inpatient workloads. This may constrain clinical encounters to prescription writing and brief lifestyle advice, with health education being substantially neglected in practice. Moreover, insufficient management of MASLD in clinical practice is not merely an issue of individual clinicians but reflects that, within current chronic disease management frameworks, MASLD is often treated as a secondary concern in non-specialist care with relatively limited management investment. A survey of hepatologists’ knowledge of MASLD showed that most hepatologists rarely screen for MASLD and only a minority recognize that MASLD can lead to severe liver disease (20). When limited professional guidance coincides with the frequent dissemination of misinformation online, patients are more likely to hesitate between contradictory views, further diminishing intervention effectiveness.

Therefore, nursing practice for postmenopausal patients with MASLD should move active health assessment to the early stages of diagnosis and follow-up. First, implement practical assessment tools to identify high-risk groups early and deliver stratified management. Second, incorporate knowledge associated with menopause to translate complex metabolic changes into patient-understandable processes and emphasize the core message that risks are real but can be improved through management. Third, establish traceable pathways to authoritative information sources and provide targeted clarification of common online misconceptions during follow-up.

### 4.2 The intersection of physical and psychological changes with family roles increases the difficulty of lifestyle modification

From the perspective of postmenopausal women, this study delineates multiple sources of resistance to lifestyle modification. In terms of physical activity, the combined effects of osteoporosis risk, knee degeneration, chronic pain, and declining physical capacity raise concerns about exercise safety, leaving some patients caught in the tension of “wanting to be active but being unable to” or “not daring to,” with activity often reduced to household manual labor. In dietary practices, long-standing habits such as high salt and oil intake, preferences for sweets, and excessive fruit consumption are further reinforced by shared family meals and a culture of social reciprocity: family dining often prioritizes others’ tastes, and social gatherings introduce pressure to “save face,” so even when a lighter diet is acknowledged as beneficial, it is difficult to sustain because of “accommodating family” or “not wanting to spoil the mood.” A study in China reported that customary practices and cultural norms can undermine the maintenance of dietary interventions, and that health education alone has limited effectiveness when family support is lacking (21, 22).

Notably, this difficulty shows clear intergenerational and gendered role patterns: many postmenopausal women juggle grandchild care, spousal care, and household chores, and self-management is often subordinated to family responsibilities. Evidence suggests that, in settings marked by traditional role expectations and common intergenerational caregiving, factors such as night-time caregiving, taking on more than 50% of caregiving tasks, and caregiving time exceeding 6 h per day are negatively associated with health self-management (23, 24). However, other scholars have proposed that caregiving at a moderate level may increase life satisfaction, reduce depressive symptoms, and be associated with improved blood pressure (25). Therefore, the influence of intergenerational caregiving on health behaviors is likely to be two-sided: when support is adequate, responsibilities are clearly shared, and intensity is moderate, it may offer emotional comfort and a sense of value; when intensity is high, night-time demands are frequent, and the burden is borne by women alone over the long term, it is more likely to hinder healthy behaviors.

From the active health perspective, simply telling patients to “eat less and move more” is unlikely to address these complex circumstances. Accordingly, interventions for postmenopausal patients with MASLD should consider both physical and psychological capacity as well as the family context. Clinicians should recognize the role of family members in disease management, provide family-oriented education, and foster a supportive home environment to improve adherence. Exercise planning should prioritize safety and feasibility, emphasizing low-impact, time-efficient, and progressively graded approaches. Dietary strategies should also extend from the individual to the family table by improving acceptability within the household and offering practical options for dining-out and social gatherings.

These findings also suggest that the challenges described in postmenopausal women should be interpreted in relation to the broader MASLD literature. Similar to the general MASLD population, postmenopausal women appear to share core self-management needs, including better disease knowledge, individualized lifestyle guidance, and sustained professional support (16). However, in the present study, these common needs were further complicated by menopause-related symptoms, emotional fluctuation, body-image concerns, osteoarticular discomfort, and family caregiving responsibilities, all of which made lifestyle modification more difficult to implement in daily life. At the same time, some barriers identified here, such as reduced physical capacity, multimorbidity, and entrenched health habits, may also reflect age-related influences rather than menopausal status alone. Therefore, age and menopausal status should be regarded as related but distinct factors in understanding MASLD self-management experiences (26).

### 4.3 Potential effects of emotional responses on self-management behaviors

This study found that emotional fluctuations were common among postmenopausal patients with MASLD during long-term self-management: some participants reported anxiety, frustration, and other negative emotions when facing weight regain, perceived failure of control, or dissatisfaction with body image, which weakened their willingness to persist with healthy behaviors and made disengagement from self-management more likely; others adopted a “let it be” stance, viewing the illness as part of aging, yet this stance often resulted in insufficient motivation, leaving health management at the level of knowledge rather than sustained action. Previous studies indicate that anxiety and depression are more prevalent in patients with MASLD than in the general population, and emotional distress not only shapes subjective judgments about disease severity but also reduces receptivity to and adherence with health education and increases the risk of behavioral discontinuation (27). Active Health Theory emphasizes that emotional experience and psychological readiness directly influence whether individuals can shift from passive adherence to active behavior (17, 18). Our findings further suggest that the menopause transition brings emotional volatility, changes in body shape, and role burden that intersect and make self-management more susceptible to emotional states, creating a cycle of “attempt-setback-interruption.” In addition, systemic inflammation may partly explain the connection between MASLD and psychological distress. The liver-spleen axis has been proposed as an immune-metabolic pathway involved in MASLD, reflecting interactions among hepatic steatosis, splenic immune activation, oxidative stress, and chronic low-grade inflammation (28). Therefore, early identification of patients with anxiety, low mood, or body image concerns is important. In follow-up practice, brief screening tools such as the PHQ-2 and GAD-2 may be used to quickly identify patients at risk of emotional distress, and positive screening results can prompt further assessment, referral, or timely psychological support. For patients with persistent low mood, marked loss of interest, suicidal ideation, or obvious functional impairment, timely referral to mental health professionals is necessary. In such cases, psychological interventions, mind–body therapies, and, when appropriate, specialist-guided pharmacological treatment should be considered together with lifestyle management rather than relying solely on general health education. At the same time, nurses can provide brief, feasible emotion-regulation guidance, such as simple breathing-based relaxation, short mindfulness exercises, or supportive communication focused on setbacks in weight control, to help patients reduce self-blame and rebuild confidence in self-management. Integrating these low-burden psychological support strategies into routine follow-up may improve emotional coping, promote sustained behavioral engagement, and ultimately enhance quality of life.

### 4.4 The motivating role of active health intention in translating self-management into practice

It is worth noting that inadequate risk perception and active intention are not mutually exclusive. Some patients demonstrate a willingness to attempt behavioral changes after receiving initial information, yet discontinue due to a lack of sustained support. Therefore, interventions should not only address cognitive biases but also promptly identify and build upon patients’ initial motivation. This study found that although some postmenopausal patients with MASLD face multiple barriers, including distorted understanding, resistance to lifestyle change, and emotional burden, they are not entirely passive. Some participants actively searched for relevant information, sought advice from peers, or identified exercise approaches suited to their own conditions through repeated trial and error; others monitored themselves using simple indicators such as changes in body weight, how tight their clothes felt, and fatigue, and spontaneously adjusted diet or activity levels. These self-directed behaviors indicate that active health intention is not absent but lacks clear professional support, which prevents it from being translated into sustainable self-management actions. Active health Theory does not frame individuals as mere “compliant recipients” but emphasizes their agency in maintaining health (17, 18). Our findings suggest that postmenopausal patients with MASLD show intention and early behavioral initiation; however, without clear goals, actionable pathways, and professional feedback, this intention often remains at the level of sporadic attempts and rarely develops into planned, paced self-management practice. Participants commonly stated that what they truly need are plans that are “specific, feasible, and sustainable,” rather than conceptual advice such as “eat less and move more,” indicating that their main difficulty lies less in willingness (“whether to do it”) than in implementation (“how to do it”). Therefore, nursing practice should leverage patients’ existing active health intention as a point of entry. First, brief conversations can be used to identify concrete behaviors that patients have already tried or are more likely to accept, and then to co-set achievable goals on that basis rather than imposing requirements. In parallel, simple channels such as phone calls or WeChat can provide intermittent yet ongoing feedback and encouragement, guiding initially intermittent attempts toward more stable behavior patterns. Future interventions that use patients’ existing intention as a fulcrum for guidance, rather than one-way delivery of demands, are more likely to achieve long-term maintenance of self-management behaviors in real-world contexts.

### 4.5 Limitations

The study also had some limitations. This study was conducted in a single Grade III Class A hospital in Nanjing. All interviews were carried out after the physical examination. Future research should expand the research locations and include patients from primary hospitals to generate more comprehensive and universal evidence. Moreover, while efforts were made to ensure accurate translation, the inherent cultural nuances in participants’ original Chinese expressions may not have been fully conveyed in English. Therefore, there is a certain degree of bias. Future studies can include populations with different levels of fibrosis risk, metabolic comorbidities and care burden in multi-center samples to test the key contextual factors proposed in this study. Secondly, family involvement interventions can be implemented and their effects can be verified through randomized controlled or mixed methods.

## 5 Conclusion

Guided by Active Health Theory, this study synthesized themes from the self-management experiences of postmenopausal patients with MASLD, including Inadequate awareness of MASLD risk, obstacles under the physical and mental burden related to menopause, gaps in the support system, bidirectional effects of emotional experiences on self-management, and active health seeking and self-motivation. The findings suggest that, amid the overlapping influences of liver disease, menopausal transition, and family roles, postmenopausal women face substantial barriers to self-management while also possessing active health potential that can be activated, offering important implications for developing targeted self-management programmes.

## Data Availability

The raw data supporting the conclusions of this article will be made available by the authors, without undue reservation.
